# Thai Hom Mali Rice: Origin and Breeding for Subsistence Rainfed Lowland Rice System

**DOI:** 10.1186/s12284-018-0212-7

**Published:** 2018-04-09

**Authors:** Apichart Vanavichit, Wintai Kamolsukyeunyong, Meechai Siangliw, Jonaliza L. Siangliw, Suniyom Traprab, Siriphat Ruengphayak, Ekawat Chaichoompu, Chatree Saensuk, Ekapol Phuvanartnarubal, Theerayut Toojinda, Somvong Tragoonrung

**Affiliations:** 1 Rice Science Center, Kasetsart University, Kamphaeng Saen, Nakhon Pathom, 73140 Thailand; 2 Agronomy Department, Faculty of Agriculture at Kamphaeng Saen, Kasetsart University, Kamphaeng Saen, Nakhon Pathom, 73140 Thailand; 3 Rice Gene Discovery Laboratory, National Center for Genetic Engineering and Biotechnology (BIOTEC), National Science and Technology Development Agency (NSTDA), Kasetsart University, Kamphaeng Saen, Nakhon Pathom, 73140 Thailand; 4 Bureau of Rice Research and Development (Rice Department), 50 Paholyothin Rd, Chatuchak, Bangkok, 10900 Thailand; 5 Plant Biotechnology Research Unit, National Center for Genetic Engineering and Biotechnology (BIOTEC), National Science and Technology Development Agency (NSTDA), 113 Thailand Science Park, 113 Thailand Science Park, Khlong Luang, Pathum Thani, 12120 Thailand; 6 National Center for Genetic Engineering and Biotechnology (BIOTEC), National Science and Technology Development Agency (NSTDA), 113 Thailand Science Park, 113 Thailand Science Park, Khlong Luang, Pathum Thani, 12120 Thailand

**Keywords:** Thai Hom Mali, *Oryza sativa* L, Marker-assisted backcross selection, Gene pyramiding, Amino aldehyde dehydrogenase, Flooding tolerance, Blast resistance, Bacterial blight resistance, Brown planthopper tolerance

## Abstract

The world-renowned Thai Hom Mali Rice has been the most important aromatic rice originating in Thailand. The aromatic variety was collected from Chachoengsao, a central province, and after pure-line selection, it was officially named as Khao Dawk Mali 105, (KDML105). Because of its superb fragrance and cooking quality, KDML105 has been a model variety for studying genes controlling grain quality and aroma. The aromatic gene was cloned in KDML105, as an amino aldehyde dehydrogenase (AMADH) or better known as *BADH2* located on chromosome 8. Later on, all other aromatic rice genes were discovered as allelic to the AMADH. As a selection of local landrace variety found in rainfed areas, the Thai Jasmine rice showed adaptive advantages over improved irrigated rice in less fertile lowland rainfed conditions. Because KDML105 was susceptible to most diseases and insect pests, marker-assisted backcross selection (MABC) was used for the genetic improvement since 2000. After nearly 17 years of MABC for integrating new traits into KDML105, a new generation of KDML105, designated HM84, was developed which maintains the cooking quality and fragrance, and has gained advantages during flash flooding, disease, and insect outbreak.

**Electronic supplementary material:**

The online version of this article (10.1186/s12284-018-0212-7) contains supplementary material, which is available to authorized users.

## Origin and distribution

Thai Jasmine rice is officially known as Thai Hom Mali rice. Hom Mali local landrace varieties were widely distributed throughout Thailand under a variety of names including ‘Khao Hom’, ‘Hom Mali’, and ‘Khao Mali’. In 1945, the best Hom Mali local variety was discovered by a farmer in Lam Pradoo district, Chonburi province, and in 1951, 199 panicles of the local variety were selected from a nearby district of Chachearngsoa province for pure line selection (Bureau of Rice Research and Development 2010). Based on its superior physical appearance, cooking quality, and grain aroma, row ‘105’ was selected and officially named “Khao Dawk Mali 4–2-105” or “Khao Dawk Mali 105” or “KDML105” for short. KDML105 was released to farmers in 1959. Thai Jasmine rice was produced annually at approximately 9.4 M ton of paddy rice from 4.3 M ha (Department of Foreign Trade 2014; Varinruk 2017).

KDML105 has a tall, lanky, floppy architecture. The average plant height at harvest is approximately 140 cm. KDML105 has a photoperiod sensitivity that determines its flowering date around October. Specifically, the critical induction signal is the day/night time duration. When day length is shorter than 11:52 h, KDML105 will initiate its floral organ (Kumboonreang 2011). The critical day length for photoperiod sensitivity can be met at the end of September when rainfall is normally heavy. Therefore, the full bloom is normally expected in the last two weeks of October, and harvesting will start in the second week of November every year. This photoperiod sensitivity is considered an adaptive advantage for lowland rainfed rice to survive and produce without irrigation water.

## Review

In traditional lowland rainfed rice cultivation, farmers ask the rice spirit to protect their crop from flooding or drought during cultivation. KDML105 and its mutagenized counterparts, RD15 and RD6, have been widely cultivated in such a stressful mega-ecosystem which occupies 70% of the rice area in Thailand (Jongdee et al. 2006). Characterized by fluctuation in rainfall distribution and poor soil fertility, the lowland rainfed area produces the highest quality Jasmine rice. Adaptation to the infertile lowland rainfed area of the northeast is the benefit of this high-quality rice. It is mildly tolerant to drought, salinity, and acid sulfate soil (Bureau of Rice Research and Development 2010). KDML105 is not a well-known drought-tolerant variety, but its adaptability in a rainfed lowland ecosystem was ascribed to the plasticity of the root system under abiotic stresses (O’Toole and Bland 1987). Plasticity describes the ability of roots to respond to soil moisture fluctuation (Banco et al. 2000). Particularly, in a paddy field with hardpan, KDML105 consistently expresses its root growth adaptability to water stress via greater root branching (Kano-Nakata et al. 2013). Expression of root-branching ability in a shallow soil layer plays an important role in capturing water for rapid recovery from drought stresses and is the key adaptive trait of KDML105 in lowland rainfed conditions (Kano-Nakata et al. 2013).

Most rice is very sensitive to salinity, at a salinity level from 3 dS m^− 1^ (USDA 2013). The salinity at such level affects rice growth by reducing germination rate, plant height, tillering, root growth, and spikelet fertility. At the EC_e_ as low as 3.5 dSm^− 1^, grain yield loss can be at about 10% depending on varietal difference (Rice Knowledge Bank, International Rice Research Institute, IRRI). Under a salt-affected area in the NE of Thailand, KDML105 was adaptive to salt-affected area in the NE, where 1.84 M ha or 16% of the lowland rainfed area is classified as an inland saline area ranging from 11 to 35 dS m^− 1^ (Im-Erb et al. 2013; Arunin and Pongwichian 2015). The saline soil in this area is high in sodium and chloride content and has a sandy soil texture, low organic matter, and minimal fertility (Arunin 1984). Cultivation of KDML105 in this area was located on slightly to moderately salt-affected soils, with ECe of 2–8 dS m^− 1^ (Im-Erb et al. 2013; Arunin and Pongwichian 2015). However, KDML105 is less productive in high (8–16 dS m^− 1^) and very high saline soil (> 16 dS m^− 1^). Therefore, increasing KDML105 salinity tolerance can help poor farmers to grow this fragrant rice in high-salinity areas.

**Fig. 1:**
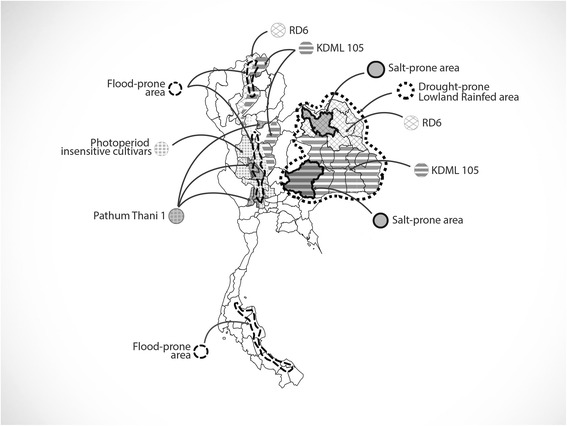
Geographical distribution of Thai Hom Mali rice, RD6, photoperiod insensitive aromatic and non-aromatic rice cultivars, in relation to abiotic stresses drought, salinity, and flooding, in the lowland rainfed area in Thailand

The plasticity of KDML105 seems to be inherited to its irradiated mutants, RD15 and RD6. These three cultivars occupied 5.5 M ha of the mega-lowland rainfed area mostly in the Northeast of Thailand (Fig. 1). On the other hand, the three mega-varieties, KDML105, RD15, and RD6, were not successful in the central plain due to the widespread occurrence of various diseases and insect pests in irrigated areas. This implies that the three Jasmine rice varieties are more adaptable to abiotic than biotic stresses. Therefore, breeding for resistance to biotic and abiotic stresses is the most sensible approach to strengthen KDML105 in lowland rainfed and irrigated areas.

## Cooking quality, fragrance, and interaction with environment

The superb cooking quality of KDML105 is characterized by its soft-texture, aromatic, long-slender white rice. Low amylose content, a low degree of gelatinization, and soft gel consistency are biochemical characteristics of the cooked KDML105 rice. When grown in its optimal environment, the physical appearance of polished grains is pearly, clear, very long and slender, with a glossy exterior. The dimension of brown rice is 2.1 × 7.5 × 1.8 mm (width x length x thickness), and the length/width ratio is 3.42. Its head rice yield is 58–60%.

### Cooking and eating quality

Eating quality is determined by consumer preference and perception of cooked rice under an optimal cooking protocol. Cooking quality can be determined as soft-texture based on physicochemical properties including amylose content (AC), amylopectin content (AP), gelatinization temperature (GT), and gel consistency (GC) (Juliano and Perez 1984; Lanceras et al. 2000; Hsu et al. 2014). QTL mapping of the cooking quality of KDML105 was reported (Lanceras et al. 2000) in a 141 F8 recombinant inbred line (RIL) population from a cross between KDML105 and CT9993–5–10-1-M, a drought tolerant, *japonica*, upland rice from the Center of International Tropical Agriculture (CIAT). The major QTL on chromosome 6, known as the Waxy locus, with modifier genes on chromosomes 3, 4, and 7, determined 14–16% AC of KDML105. AC and GC were linked on QTLch6. The waxy allele of KDML105 was classified as *Wx*^*b*^ or *Wx*^*g1*^ (Lanceras et al. 2000; Teng et al. 2012). Three QTLs for GT were mapped on chromosome 6 as the single major QTL, and the two minor QTLs were mapped on chromosome 2. The major QTLch6 for GT is known as the *alk* locus encoding soluble starch synthase IIa (*SSIIa*) (Umemoto et al. 2002). Grain aroma is controlled by a single recessive gene located on chromosome 8 (Singh et al. 2007; Tragoonrung et al. 1996). QTL analysis of grain aroma identified one major QTL controlling grain aroma mapped close to RG28 on chromosome 8 (Ahn et al. 1992) in Azucena (Lorieux et al. 1996), Basmati (Singh et al. 2007) and KDML rice (Tragoonrung et al. 1996). Because sensory evaluation of grain aroma is inaccurate, the discovery of genes responsible for grain aroma for MAS is the key to improving KDML105.

### Discovery of the aromatic gene

The content of 2-acetyl-1-pyrroline (2AP) is the major determinant of grain aroma, as the 2AP content and the QTL of grain aroma were coincidentally mapped on the same locus on chromosome 8 (Lorieux et al. 1996). Map-based cloning of the aromatic genes in KDML105 was initiated on *Os2AP* isogenic lines differing in 2AP content and developed from a KDML cross (Vanavichit et al. 2004; Vanavichit et al. 2005). The responsible gene, named *Os2AP*, was identified as a member of gamma-aminobutylaldehyde dehydrogenase (*AMADH*) and functions as the metabolic switch between γ-aminobutylaldehyde (GBAL) and gamma-aminobutyric acid (GABA) in non-aromatic isogenic lines (Vanavichit et al. 2005). The crystallization of the 503-amino acid *Os2AP* protein from the non-aromatic isogenic line was reported (Kuaprasert et al. 2011). The structure was predicted to contain three binding domains: an NAD+ binding, an oligomerization, and a substrate binding domain where an aldehyde dehydrogenase cysteine subdomain was located (Chen et al. 2008; Wongpanya et al. 2011). In the aromatic isogenic lines and KDML105, the specific 8-bp deletion was identified within exon 7 of the *AMADH* of KDML105, causing a premature stop codon, non-sense-mediated decay, and non-functioning of *Os2AP*, respectively (Vanavichit et al. 2005). RNAi against the Os2AP in the non-aromatic Japonica rice cv. Nipponbare resulted in the suppression of *AMADH* expression, resulting in the accumulation of 2AP at the level of KDML105 under the same growing condition (Vanavichit et al. 2005). Similar conclusions for the gene responsible for emerging aromatic rice and its aromatic compound biosynthesis switch were reported by most of the research groups as *BADH2* (Bradbury et al. 2005; Chen et al. 2008; Sun et al. 2008; Shi et al. 2008; Bradbury et al. 2008). Comparing the aromatic isogenic line and RNAi in the N^15^ dilution experiment and inclusion of the inhibitors of enzymes in polyamine biosynthesis revealed ornithine as the initial amino acid precursor of N, and polyamine is a major biosynthetic pathway leading to 2AP accumulation (Vanavichit et al. 2005; Vanavichit and Yoshihashi 2010).

### Variation in aromatic quality

Among major aromatic rice cultivars, although they carry the same aromatic allele of *AMADH,* considerable variation in 2AP content was reported, for example, in Basmati rice (0.34 ppm), Jasmine rice (0.81 ppm) and Texmati rice (0.53 ppm) (Goufo et al. 2010; Gaur et al. 2016). Environmental factors will always have a strong influence on the synthesis and degradation of 2AP content, resulting in quantitative variation of 2AP in rice grains. Additionally, other volatile compounds may add flavors to the pandan-like aroma. Analysis of volatile aromatic compounds during grain ripening identified odor-active compounds (OAC), including 2AP, decanal, pentanal, phenylacetaldehyde, hexanal, (E)-2-nonenal, nonanal, heptanal, 1-octanol, 1-octen-3-ol, and 2-pentylfuran as contributors toward the unique sensory of specific rice fragrance (Hinge et al. 2016). Also, terpenoids, another class of volatile aromatic compounds found mostly in herbs, were identified in rice bran from purple, red, and brown rice varieties (Chumpolsri et al. 2015). Also, in the rice bran extracts of KDML105, major terpenoid odorants were found, such as limonene, trans-b-ocimene, b-cymene, and linalool (Chumpolsri et al. 2015). This finding may help explain the difference in the sensory evaluation of several aromatic rice varieties before and after cooking.

### Geographical dependency

The accumulation of 2AP and grain qualities of aromatic, low-amylose rice is influenced by season and production area. The first report came from seven KDML rice samples collected between 2000 and 2001 from the north (N), central (C), and northeast (NE) regions of Thailand, which were quantified for grain 2AP content (Yoshihashi et al. 2004). The outcomes showed that the lowland rainfed area in the NE was the optimal environment for the accumulation of 2AP in rice grain (Yoshihashi et al. 2004). In particular, the NE had the best rice quality regarding physical appearance and cooking qualities (Anun et al. 2000). Environmental variation of cultivated areas affects the nitrogen, lipid, starch, fiber, and ash of polished rice (McCall et al. 1953), and low-amylose rice was particularly sensitive (Asaoka et al. 1989). Based on physical and cooking quality evaluation, the production area of KDML105 was classified as most favorable, favorable, or less favorable (Anun et al. 2000). Based on pasting properties rapid visco analysis (RVA) and the colorimetric properties of rice starch (Differential Scanning Colorimetry analysis), KDML105 environmental quality was classified into five groups: most favorable (one group), favorable (one group), and less favorable (three sub-groups) (Pitiphunpong and Suwannaporn 2009). In the most favorable area, “Tung-kula-rong-hai,” which comprises three provinces in central NE Thailand, was praised for the geographical distinction for overall Jasmine rice qualities, in particular, 2AP (Yoshihashi et al. 2004). Poor fertility, sandy and mildly saline soil, and a cool, dry, sunny atmosphere during the ripening stage are distinctive characteristics of the area that affect both grain quality and grain 2AP content (Yoshihashi et al. 2004).

## Susceptibility to biotic stresses

KDML105 and its induced-mutation RD15 and RD6, are highly susceptible to bacterial leaf blight (BLB), leaf/neck blast and brown planthopper/whiteback hopper because of the lack of R genes (Toojinda et al. 2005). Overall, the 3.2 M ha production area for KDML105 and RD15 is considered the largest single varietal type in a lowland rainfed ecosystem and is at high risk of the BLB, BL, and BPH outbreak. In lowland rainfed conditions, blast disease caused by *Pyricularia grisea* has threatened KDML, RD15, and RD6 productivity at every stage of growth, but particularly during the ripening stage, called neck blast. Of all rice growing countries, Thailand has the most diversified pathotypes in the world (Sreewongchai 2008; Chaipanya et al. 2017), as the fungus mutates quickly. In October 1993, 200,000 ha of Jasmine rice was devastated during the ripening stage, when farmers have no option for mitigation (Rice Today January-March 2006). Breeding for durable blast resistance from various donors with conventional and marker-assisted selection has reduced farmers’ risk of devastation by disease and ensured the grain yield and quality of Thai Jasmine Rice. Lacking major R genes, KDML and RD15 are highly susceptible to BPH (*Nilaparvata lugens* Stål) in every stage of plant development (Jairin et al. 2005). Also, releasing aromatic compound 2AP from all plant parts making KDML even more risk of being devastated as 2AP may act as a possible chemical attractant to brown planthopper (BPH) (Kamolsukyunyong et al. 2013). Therefore, breeding for durable resistance to BLB, BL, and BPH of KDML is the most practical mitigation strategy to prevent crop loss.

## New generation of jasmine Rice: Plus one

At the initial stage, mutation breeding utilizing gamma rays generated two important progenies: RD6, waxy Jasmine rice, and RD15, early-maturing Jasmine rice, which were released in 1977 and 1978, respectively (Rice Department). The first blast tolerant variety with a Jasmine-like quality was initiated in 1999 using IR77924–62–71-1-2 as the donor for two backcrosses to KDML, followed by pedigree selection (Supapoj et al. 2009). The elite progeny RD33, photoperiod-insensitive Jasmine rice, was released in 2006, as an alternative aromatic variety with blast resistance for NE and N Thailand (Rice Today Jan-March 2006). Marker-assisted backcrossing was comprehensively developed to add more resistance genes for diseases, insects, and abiotic stresses to KDML105 (Toojinda et al. 2005). These new generations of KDML progeny possess new traits, such as tolerance to submergence, bacterial leaf blight, and brown planthopper (Table 1). Background selection was performed using 65 well-distributed SSR markers to preserve most of the KDML105 genetic background for the adaptability and market quality of the Thai Jasmine rice (Table 2).

**Table 1:** KASP SNP markers for specific gene target used for MABC for improving KDML105

Traits	Marker Name	Chr.	QTL/gene	LGC code^f^
Aroma	Aroma_2-3^e^	8	*OsBADH2*	002–0829.1
Amylose content (AC)	wx_5UTR_G/T^a^	6	*GBSS*	002–0052.1
Gelatinization temperature (GT)	ALK_ex8_SNP_GC/TT^a^	6	*SSIIa*	002–0049.1
Bacterial leaf blight (BLB)	xa5^a^	5	*xa5*	002–0775.1
Bacterial leaf blight (BLB)	SNP_P100 Xa21^e^	11	*Xa21*	002–0998.1
Brown planthopper (BPH)	OsSTPS2_21bp_del^b^	4	*OsSTPS2*	002–0120.1
Brown planthopper (BPH)	OsLecRK3_QBPHR^e^	4	*OsLecRK3*	002–0263.1
Brown planthopper (BPH)	Bph32_2_1223332^e^	6	*BPH 32*	
Blast (BL)	BLch11-Pikm-2^c,d^	11	*NB-ARC domain containing protein*	002–0754.1
Blast (BL)	TBGI055578_TC_Chr1^a^	1	*secretory carrier-associated membrane protein*	002–0768.1
Blast (BL)	TBGI055841^a^	1	*hypothetical protein*	002–0819.1
Blast (BL)	TBGI453126^a^	11	*NBS-LRR disease resistance protein*	002–0820.1
Blast (BL)	TBGI453598^a^	11	*retrotransposon protein*	002–0821.1
Blast (BL)	TBGI454069^a^	11	*receptor kinase-like protein*	002–0822.1
Blast (BL)	TBGI454717^a^	11	*C2H2 zinc finger protein*	002–0823.1
Blast (BL)	TBGI454800^a^	11	*zinc finger, C3HC4 type*	002–0824.1
Submergence (SUB)	Sub1A_SNP1^e^	9	*Sub1A*	002–0152.1
Submergence (SUB)	Sub1C_loci5^a^	9	*Sub1C*	002–0995.1

^a^ Ruengphayak et al.; 2015

^b^ Kamolsukyunyong et al.; 2013

^c^ Chaipanya et al. 2017

^d^ Ashikawa et al.; 2012

^e^Uupublished

^f^ LGCgroup, 2015

**Table 2:**
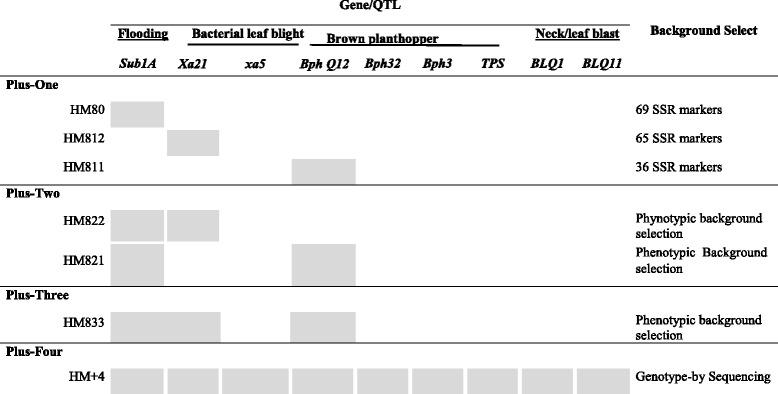
Pyramiding nine target genes/QTLs into the KDML105 background by marker-assisted backcrossing illustrated in Fig. 2. Background selection with markers was conducted only in the Plus-1 and Plus-4 BILs. X= The gene/QTLs were added to the new varieties by backcrossing

*TPS* Terpene synthase (OsSTPS2)

### KDML-Sub1

**Fig. 2:**
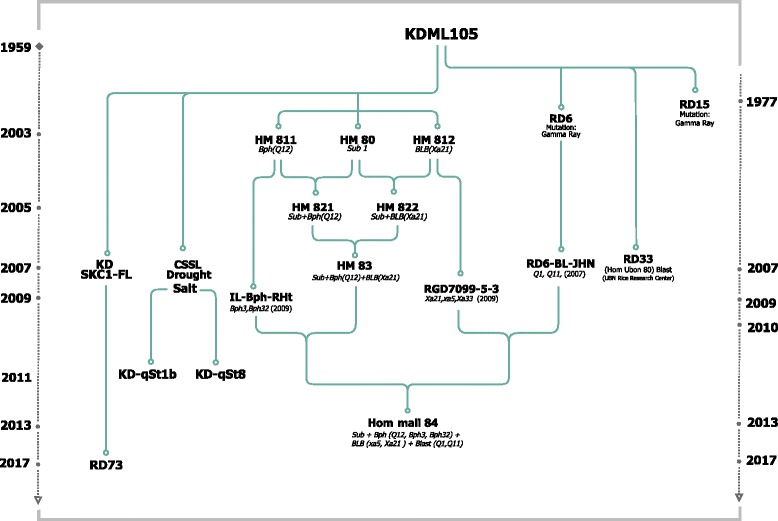
Schematic development of KDML105 from a well-adapted, local aromatic rice cultivar to new KDML-like rice varieties with resistance to submergence, bacterial leaf blight, leaf/neck blast, and brown planthopper using comprehensive marker-assisted gene/QTL pyramiding backcross breeding

The first marker-assisted selection line generated, HM80, was the first *Sub1*- Jasmine rice with flash flooding tolerance using IR49830–7–1-2-2 as the donor (Siangliw et al. 2003). This line was officially released in 2013 by the Rice Department. After the worst flooding in decades that affected Thailand in 2011, RD51 (HM80) was released for farmers in 2013. The planting area of the RD51 in 2013–14 was only 3000 ha in a flood-prone area or only 1% of the Thai Jasmine rice (KDML105 + RD15) (Table 3). Later, the HM80 was used as the general flooding donor to develop advance backcross pyramid lines (Fig. 2, Table 4).

**Table 3:** Planting area of the Thai Jasmine rice (KDML105 + RD15), KDML-derived varieties, RD6, RD51, RD33, and Pathumthani 1 (PTT1), in the wet and dry seasons, 2013–14 (Unpublished Rice Department 2014)

Varieties	Area (ha)		Total (ha)
	Wet season	Dry season	
KDML105	3,870,560	3630	3,874,190
RD15	238,470	1580	240,050
RD6	1,407,250	390	1,407,640
RD51	3170	290	3460
RD33	110	2950	3060
PTT1	8900	46,110	55,010
Total (ha)	5,528,460	54,950	5,583,410

**Table 4:** Trait evaluation, agronomic characteristics, grain quality, and cooking quality traits of the four selected HomMali 84, KDML105, and KD EDVs (For trait evaluation methods, see Additional file 1)

Name	HM84 progenies				KDML105	KD EDV			
						KD-BLB	Plus III	RD6-Blast	KD-Rathu
	6_14G08	24_16D06	2_14A03	4_14B09					
^a^Sub	84.67	55.00	49.00	63.67	7.00	5.3	75.7	33.3	6.5
BLB(4 isolates)	R	R	R	R	S	R	R	S	S
BPH (2 biotype)	R	R	R	MR	S	S	MR	S	R
Blast (8 Mixed)	R	R	R	R	S	S	S	R	S
^b^DM	126.67	125.33	129.00	120.33	120.67	120.33	122.00	121.67	127.30
NTP	13.00	10.00	12.33	13.67	14.33	9.00	13.67	8.33	12.67
PH(cm)	112.80	114.77	112.10	113.97	98.23	101.33	113.33	110.23	99.77
PSF(%)	72.33	72.27	71.07	68.57	68.10	60.27	77.27	55.07	74.33
TGW(g)	30.03	29.93	27.80	27.90	29.93	29.65	28.89	26.33	27.97
GY(kg/ha)	2766.69	2887.50	2266.69	2833.31	2554.19	2360.44	3130.31	2795.81	2921.06
^c^BR1/ (%)	76.06	73.39	68.10	73.03	70.40	70.64	69.48	72.54	71.88
HR1/ (%)	62.08	61.26	55.02	49.00	57.84	50.44	51.49	58.14	58.85
GL/W1/	3.4	3.16	3.2	3.29	3.42	3.24	3.22	3.11	3.15
PRL1/ (cm)	0.75	0.73	0.75	0.78	0.72	0.7	0.71	0.7	0.67
ASV1/	6.0	6.1	6.0	6.0	6.0	2.3	6	6.1	6.2
Amylose(%)	16.42	17.7	14.14	14.55	17	16.58	17.21	8.2	16.94

^a^/Trait evaluation

Sub = Average % plant survival (%PS) after 15 days of flash flooding

BLB = average lesion length in centimeters of the damage caused by the BB isolate including TXO152, TXO85, TXO155 and TXO156

BPH = Severity scores with UBN biotype at 9 DAI when TN1, the susceptible control died

Blast = Average blast injury score when attacked by 8 mixed blast isolates from Thailand

^b^/Agronomic characteristics

DM (days to maturity), NTP (number of tillers per plant), PH (plant height from the soil surface to the neck of the panicle), PSF (percent spikelet fertility), TGW (1000-grain weight) and GY (grain yield)

^c^/Grain quality and cooking quality

BR (brown rice), HR (head rice), GL/W (grain length-width ratio), PRL (polished rice length), AC (amylose content; Julaino, 1971), ASV (alkaline spreading value)

EDV = Essentially-derived variety

### KDML-Xa21

The introduction of the *Xa21* gene from the highly resistant donor line, IR1188, into KDML105 successfully generated the first Jasmine-like BLB resistant line, HM812 (Fig. 2, Table 4). Because of the limitations of *Xa21* against multiple strains of *Xanthomonas oryzae (Xo)* found in Thailand, *xa5*, *xa33* (t), *xa34*(t), and qBB11 were introduced into the KDML105 background by MABC (Korinsak, 2009).

### KDML-BphQTLCh12

More than 30 *BPH* R genes/quantitative trait loci (QTL) were mapped (Ling and Weilin 2016). Eight *BPH* R genes have been identified, including *BPH14* (Du et al. 2009), *BPH26* (Tamura et al. 2014), *BPH3* (Liu et al. 2014), *BPH29* (Wang et al. 2015), *BPH9* (Zhao et al. 2016), *BPH18* (Ji et al. 2016), *BPH32* (Ren et al. 2016), and *BPH31* (Prahalada et al. 2017). After the rigorous screening of the rice germplasm, two broad-spectrum resistant donors for BPH were identified. Abhaya (Aba), a gall midge-resistant variety from India (Kalode et al. 1993; Rao and Kandalkar 1992), and Rathu Heenati (RHt), a broad-spectrum BPH-resistant variety from Sri Lanka (Lakshminarayana and Khush 1977), were used as donors in MABC to introduce durable BPH resistance into KDML. At the initial phase, the MABC program successfully introduced the *BphQTLCh12* donated from Abhaya into HM811 (Table 4)*.*

## New generation of jasmine Rice: Plus-two and plus-three

Based on HM80 (*Sub1*) and HM811 (*BPHch12*), the major QTL for flooding tolerance, *Sub1*, was pyramided into HM811 using MABC with extreme phenotypic selection (Korinsak et al. 2016). The first pyramid line, HM821*,* gained Jasmine-like physical, fragrant and cooking qualities with enhanced submergence tolerance and BPH resistance (Fig. 2). The second Plus-2 line, HM822 (*Sub1A* and *Xa21*), was combined by backcrossing HM80 to HM812 (*Xa21*). Following the similar MABC platform, HM821 was backcrossed to HM822 to generate the Plus-three line, HM83*,* in 2007 (Fig. 2).

## New generation of jasmine Rice: Plus-four

Significant improvements to HM84 from the HM83 generation included introduction of new BPH, BLB, and Blast (BL) resistance into the HM83 background. This was the first generation into which the two QTLs for resistance to leaf and neck blast were introgressed into the Plus-3 background. In total, nine foreground genes/QTLs were introgressed into four Plus-4 BILs (Tables 2 and 4).

### Integration of durable RHt-BPH

HM811 was utilized in several pyramiding breeding experiments until the Abhaya-derived *BphQTLch12* was no longer effective against the emerging biotypes of BPH. To overcome such rapid evolution, a more durable, tolerant donor, Rathu Heenati (RHt), was used for MABC in the second phase*.* QTL mapping analysis of RHt revealed *BphQTL chr4* (Sun et al. 2005) and *BphQTLchr6* (Jairin et al. 2007), which are later referred to as *Bph3* (Liu et al. 2014) and *Bph32* (Ren et al. 2016), respectively. Introgression of the QTL from RHt, a high-amylose rice, into the soft, susceptible Jasmine rice background was a challenge because of the tight linkage between *BphQTLch6* and the waxy gene (GBSS). The genetic distance between RM589 (*BphQTLch6*) and RM190 (*Waxy*) is 1.6 cM (Jairin et al. 2007) or 383 Kb (Jairin et al. 2009). The first step to break the tight linkage between the two loci was to generate a large F2 population between KDML105 (*waxy b, bphQTLch6*) and RHt (*Waxy A, BphQTLch6*), followed by rigorous MAS for the (*waxy b, BphQTLch6*) haplotype (Jairin et al. 2007). The recombinants were backcrossed to KDML by MABC with ฺBPH screening using a broad spectrum of Thai BPH biotypes (Jairin et al. 2007). For *BphQTLch4*, one resistant IL-BPH carried a functional sesquiterpene synthase (STPS) gene located on the long arm of chromosome 4. STPS was up-regulated by BPH infestation in the IL-BPH but with no expression in infested KDML, which carries a large mutation in its promoter region (Kamolsukyunyong et al. 2013). Analysis of monoterpenoid profiling released upon infestation from the IL-BPH and KDML revealed the differential accumulation of (E)-citral, citronellal, (E)-geraniol, β-citronellol, citronellyl acetate, and geranyl acetate in the BPH resistant IL (Pitija et al. 2014). Also, during BPH infestation, KDML105 activated the amino acid-mediated pathway, while IL-BPH-RHt activated nucleotide biosynthesis by the purine and pyrimidine compound-mediated salvage pathway (Uawisetwathana et al. 2015). Such targeted release of specific terpenoids and amino acids upon insect infestation formed the basis of antibiosis and antixenosis of the interaction between rice and BPH.

### Broad-spectrum blast resistance

Leaf and neck blast QTLs were genetically mapped using two recombinant inbred line (RIL) populations derived from KDML x JHN (Noenplab et al. 2006) and JHN x IR64 (Sreewongchai et al. 2010). The two broad-spectrum resistant QTLs derived from JHN, *BLQTLch1_JHN* and *BLQTLch11_JHN,* contributed 35–43% percent variance explained (PVE) using three blast isolates. They were backcrossed using markers into RD6 (RD6-BL-JHN) (Wongsaprom et al. 2010) and were used as the donor for HM83. Gene cloning of the two QTLs revealed *Pish_J* and *Pi7_J* as BLQTLch1_JHN and BLQTLch11, respectively (Chaipanya et al. 2017). Four *BLQTLs* were combined into RD6 (RGD7005–164) (Noenplab et al. 2006; Wongsaprom et al. 2010; Sreewongchai et al. 2010; Korinsak 2010).

### Strengthening of BLB resistance

With the rapid evolution of *Xo* pathotypes in recent years, screening of R genes for broad-spectrum resistance against the pathotypes in Thailand identified *xa5* and *Xa33* as stronger R genes compared to *Xa21* (Korinsak 2009). By using five effective *Xo* isolates, pyramided backcross inbred lines (BIL) with the combination of *xa5*, *Xa21*, *xa33*(t), *Xa34*(t), and *qBB11* on an indica variety were more effective than BIL with single R genes. Among single R genes, *xa5* showed the broadest resistance followed by *Xa34*(t) > *xa33*(t), > *Xa21* (Korinsak 2009). The addition of *xa5* to HM83 significantly improved resistance to BLB in the lowland rainfed and irrigated areas of Thailand. Therefore, deploying the best combination of R genes may prolong the productivity of newly-developed BLB resistant KDML105 in irrigated area.

### Successful pyramiding

HM84, the outcome of genetic integration of the three KDML plus-one lines, delivered stronger BPH, BLB, and BL resistance into HM83 (Fig. 2 and Table 4). Nine foreground genes/QTLs were incorporated by MABC into KDML105 in a stepwise approach, from Plus-1, Plus-2, Plus-3, and Plus-4 (Table 2). Four sub-lines of HM84 derived after background selection showed tolerance to submergence, BPH, BLB, and BL, while good cooking quality and aroma were maintained (Table 4). Under pest-free conditions, HM84 on average yielded almost equal to KDML105 (Table 4). Under flooding, BLB, BL, and BPH epidemics, HM84 showed clear advantages over the susceptible KDML105 due to the addition of resistance genes/QTLs (data not shown).

## KDML-CSSL as a platform for improving drought tolerance

The limitation of map-based cloning of QTL had been the driving factor in the development of KDML105 chromosome segment substitution lines (CSSLs). The process of map-based cloning requires the development of near-isogenic lines for fine-scale mapping followed by QTL cloning. CSSL is a novel mapping population that carries a specific chromosomal segment from a donor line in the genetic background of the recurrent line. Association of QTL with a particular chromosomal segment can be performed by genetic analysis using a CSSL population; at the same time, CSSLs can be developed quickly as NIL-containing target regions/QTLs of interest. This is a good strategy to accurately study genetic regions for complex traits such as drought resistance.

A population of chromosomal segment substitution lines with a KDML105 background was developed by crossing KDML105 with IR68586-F2-CA-31 (also known as DH103) and IR68586-F2-CA-143 (DH212), which are doubled haploid lines derived from a cross between CT9993–510–1-M (CT9993), an upland japonica rice, and IR62266–42–6-2 (IR62266), an irrigated indica rice (Toojinda et al. 2011). The QTL segment in the CSSLs was originally mapped from the doubled haploid population of CT9993 and IR62266. These populations differ in potential yield, osmotic adjustment (OA), and root characteristics such as a deep, thick rooting system. This population was developed at CIAT, Colombia, and the IRRI, Philippines. Several research institutes have collaborated and used this population for the genetic study of traits associated with drought tolerance (Blum et al. 1999; Tripathy et al. 2000; Zhang et al. 2001; Babu et al. 2003; Robin et al. 2003; Nguyen et al. 2004).

The QTL study was conducted by phenotyping the 220 DH lines and parents with yield, yield components and agronomic traits under control and drought conditions. A population of 154 DH lines was randomly selected from the full set used in developing the genetic map (Zhang et al. 2001), which was used to identify the QTL-controlling traits above. Five chromosomal segments, namely, chromosomes 1, 3, 4, 8 and 9, were identified carrying several overlapping QTL for drought resistance traits covering 49, 15, 53, 60 and 30 cM of the chromosomes, respectively (Lanceras et al. 2004). These regions were introgressed into KDML105 by MABC and selfing until BC_3_F_3_, and lines carrying the QTL segments were selected using SSR markers in every generation (Siangliw et al. 2007). Further backcrossing was advanced into BC_5_, and purification for homozygosity at the QTL regions was accomplished in the F_2_, F_3,_ and F_4_ generations. In total, 135 KDML105 CSSLs were developed, comprised of 30, 22, 41, 31 and 15 lines carrying QTL in chromosomes 1, 3, 4, 8 and 9, respectively (Toojinda et al. 2011; Kanjoo 2012). These isoQTL lines can be used to develop KDML Plus-5 for greater drought tolerance shortly.

## Adapting KDML to high-saline soil

The *SKC1* gene on chromosome 1, an HKT-type transporter (*OsHKT1;5*), was identified as a controller of shoot K content related to salinity tolerance in rice (Gregorio et al. 2002, Lin et al. 2004; Ren et al. 2005; Thomson et al. 2010). Introgression of *SKC1* from FL496 (IR66946-3R-196-1-1) or FL530 (IR66496-3R-230-1-1) into KDML105 was accomplished using MABC to generate KD-SKC1-FL (Fig. 2). The improved KD-SKC1-FL showed greater tolerance, with a lower Na/K ratio and higher yield under salt stress at 10–12 ds m^− 1^ (Punyawaew et al. 2016). One elite line was released to farmers under the name “RD73” by the Thailand Rice Department in 2017. In addition to *SKC1*, qSt1b, located on the lower region of chromosome 1, significantly improved photosynthesis efficiency with less injury under salinity stress (Siangliw et al. 2014; Kanjoo et al. 2011; Thomson et al. 2010; Chutimanukul et al. 2013). The third QTL, qSt8, identified on chromosome 8, protected rice plants from salinity stress. KD-CSSL-qST1/8 carrying qSt1b and qSt8 shows more tolerance under salt stress at 10–14 dS m^− 1^ (Kanjoo et al. 2011, Chutimanukul et al. 2013, Nounjan et al. 2016). Therefore, pyramiding of *SKC1*, qSt1, and qSt8 may enhance productivity under salt stress.

## Conclusions

The 58 years-old KDML105, selected from a widely adapted aromatic landrace found in the lowland rainfed area, has become an iconic rice cultivar since 1959. To continue its productivity into the next century, KDML was rigorously and carefully enriched for new traits using mutation, conventional breeding, and MABC while conserving its superb cooking quality and adaptive advantages in the lowland rainfed area. After 13 years of MABC, new generations of KDML backcross inbred lines were carefully developed by pyramiding six QTLs with six gene-specific alleles into KDML105. Salt tolerance KDML Plus-1 and Drought tolerance CSSLs are integrating into the HM84. This newly emerging HM84 will have advantages over KDML105 in that affected area where bacterial leaf blight, blast, brown planthopper, and flooding are problems. With these adaptive advantages and superb cooking quality, the innovative KDML 105 will be more productive in the subsistence and pesticide-free lowland rainfed area.

## Additional file

Additional file 1: Evaluation of abiotic and biotic stress traits and recording of important agronomic traits method. (DOCX 22 kb)

## Abbreviations

2AP: 2-acetyl-1-pyrroline
AC: amylose content
AP: amylopectin content
BL: blast
BLB: bacterial leaf blight
BPH: brown planthopper
C: Central
CSSLs: chromosome segment substitution lines
EDV: Essentially-derived variety
GC: gel consistency
GT: gelatinization temperature
HM: Hom Mali
KDML105: Khao Dawk Mali 105
MABC: marker-assisted backcross selection
N: North
NE: North-East
PVE: percent variance explained
RHt: Rathu Heenati
RIL: recombinant inbred line
RVA: rapid visco *analysis*
Xo: *Xanthomonas oryzae*
