# Supplementary material for: Thai Hom Mali Rice: Origin and Breeding for Subsistence Rainfed Lowland Rice System
Source: Rice (N Y). 2018 Apr 9;11:20. doi: 10.1186/s12284-018-0212-7 (PMC5891439; doi:10.1186/s12284-018-0212-7)
Supplement: Supplementary file 1 — Evaluation of abiotic and biotic stress traits and recording of important agronomic traits method. (DOCX 22 kb) [file 12284_2018_212_MOESM1_ESM.docx]

**Additional file 1**: Evaluation of abiotic and biotic stress traits and Recording of important agronomic traits method

**Evaluation of abiotic and biotic stress traits**

***Submergence screening***

The parents and Hom mali84 progenies were screened for submergence resistance traits. The experiment was conducted under complete submergence in an outdoor lagoon located at the Rice Science Center, Kasetsart University, Kamphaeng Sean Campus, Thailand, during the dry season of 2013. The experiment was arranged using a randomized complete block design with three replications. Sixteen three-week-old Hommali84 progenies seedlings and controls including KDML 105, KD-BLB, Plus III, RD6-Blast and KD-Rathu were transplanted in three replicate plots (plot size: 0.75 x 0.75 m^2^) at a spacing of 25 cm x 25 cm. Two weeks after transplanting, the number of seedlings was counted in each plot; then, the lagoon was filled with water to a depth of 2 m. To impose severe submergence stress, the seedlings were completely submerged for 15 days; the water level was maintained at 1–1.2 m above the leaf tip throughout the experimental period. After this period, the lagoon was drained, and the seedlings were re-exposed to air for 10 days **(Jantaboon et al. 2011; Ruengphayak et al. 2015)**. The number of surviving plants was recorded. The percentage of survival (PS) was calculated using the following equation:

PS= Number of surviving plants × 100

Total number of plants

***Bacterial leaf blight screening***

The *Xoo* isolate TXO152, TXO85, TXO155 and TXO156 were selected for this experiment. The isolate was grown following the method described by **Win et al. (2012)**. The parents and Hommali+4 were grown in a greenhouse for 30 days before inoculation. The inoculation procedures used were adapted from those described by **Korinsak et al. (2009a,b)**. Three to four fully expanded leaves of each plant were inoculated. Lesion length (LL) was measured at 12-14 days after inoculation. The resistance reaction was classified as resistant (R), moderately resistant (MR), moderately susceptible (MS) and susceptible (S) when the values of LL were 0–3 cm, 3.1–6 cm, 6.1–9 cm and more than 9 cm, respectively **(Yang et al. 2003; Lin et al. 1996)**.

***Leaf blast screening***

Thailand *Magnaporthe oryzae* 8 mixed isolates (Rice Gene Discovery, Thailand, unpublished), were used in leaf blast screening experiments. The inoculum was prepared and the plants were inoculated following the method described by **Marchetti et al. (1987)** with some modifications. Hommali+4 and their parents were grown in polyvinyl trays containing paddy field soil (four seedlings per line) following a three-replication completely randomized design (CRD). The seedlings were maintained in a greenhouse for 17 days before inoculation, after which they were inoculated with mixed isolates. Disease scoring was recorded at seven days after inoculation on a 0 to 6 scale following the procedure described by **Roumen et al. (1997)** and **IRRI (2002)**. The average score of each line was computed from the disease score measured for 12 individual plants.

***Brown planthopper (BPH) screening***

A set of Hommali84 and their parents were screened for resistance against BPH using standard seedbox screening (SSBS); the BPH population used was collected from Ubon Ratchathani provinces **(Jairin et al. 2007)**. The SSBS was conducted at the seedling stage (10days old) under greenhouse conditions following the method described by **Heinrichs et al. (1985)**. Damage scores were recorded when the susceptible control, ‘TN1,’ died (9 days after infestation; 9DAI), using the standard evaluation system **(IRRI 1996)**.

**Recording of important agronomic traits**

Traits measured included days to maturity (DM), number of tillers per plant (NTP), plant height (PH), percent spikelet fertility (PSF), 1,000 grain weight (TGW) and grain yield (GY); these traits were measured for rice plants grown during field trials at Kasetsart University, Kamphaeng Sean, Nakhon Pathom, Thailand.

Twenty-one-day-old seedlings were transplanted in three replicates in 1 x 2 m^2^ plots using 25 x 25 cm^2^ plant spacing. Agronomic traits were recorded for five randomly selected plants grown in each plot. The NTP, PH and DM were measured at maturity, and the results were averaged from five randomly selected plants in each plot. PH was measured from the soil surface to the neck of the panicle. To measure the GY in each plot, only the inner rows (containing 21 plants) were used. Two border rows on each side of the plot and the border plants of each row were discarded. The GY recorded for each plot was adjusted to 14% moisture content and then extrapolated to units of kg per ha. TGW measurements were replicated three times. Statistical analysis was performed using the STATGRAPHICS plus 3.0 software package **(Manugistics 1997)**.

**Evaluation of grain quality**

Grain quality was evaluated using grain harvested from the trials field. Rice grains of the Hommali84 and their parents were harvested at physiological maturity and sun-dried in a greenhouse. The dried grains were stored at room temperature for one month prior to the grain quality traits evaluation. Three hundred grams of grains was sampled from each replicate. The grains were mechanically dehulled and polished using a mini-polisher. Four physical grain qualities, including percentages of brown rice (BR), head rice (HR), grain length (GL/W) and %cooking elongation increased (CE), were evaluated for the polished rice. Ten grains of paddy rice were measured using a Vernier caliper, and the GL/W ratio was calculated. The polished rice grain length (PRL) was measured using the same method. The cooking elongation of the polished rice was determined by boiling 20 grains in 5 ml of dH_2_O for ten minutes. Cooked grain lengths were measured after air-drying the grains for 1 hour. Two chemical grain qualities, amylose content (AC) and gel temperature (GT), were evaluated following the procedures described by **Lanceras et al. (2000)**. GT is an indicator of the time required for cooking. The GT was indirectly estimated based on the alkali spreading value (ASV); higher values of ASV represent increased spreading in alkali and therefore represent lower values of GT; conversely, smaller values of ASV indicate higher values of GT.

**References**

Heinrichs EA, Medrano FG, Rapusas HR (1985) Genetic evaluation for insect

resistance in rice. IRRI, Los Baños, Philippines.

IRRI (1996). Standard Evaluation System for Rice. IRRI, Manila, Philippines.

IRRI (2002) Standard Evaluation System for Rice (SES). International Rice Research Institute, Los Banos, Philippine

Jairin J, Phengrat K, Teangdeerith S, Vanavichit A, Toojinda T (2007) Mapping of a broad-spectrum brown planthopper resistance gene, *Bph3*, on rice chromosome 6. Mol Breed 19: 35-44.

Jantaboon J, Siangliw M, Im-mark S, Jamboonsri W, Vanavichit A, Toojinda T (2011) Ideotype breeding for submergence tolerance and cooking quality by marker-assisted selection in rice. Field Crops Res 123(3):206-213.

Korinsak S, Sirithanya P, Toojinda T, (2009a) Identification of SSR markers linked to a bacterial blight resistance gene in rice cultivar ‘Pin Kaset’. KKU Res J (GS) 9:16–21.

Korinsak S, Sriprakhon S, Sirithanya P, Jairin J, Korinsak S, Vanavichit A, Toojinda T, (2009b) Identification of microsatellite markers (SSR) linked to a new bacterial blight resistance gene *Xa33* (t) in rice cultivar ‘Ba7’. Maejo Int J Sci Technol 3:235–247.

Lanceras J, Huang ZL, Naivikul O, Vanavichit A, Ruanjaichon V, Tragoonrung S (2000) Mapping of genes for cooking and eating qualities in Thai jasmine rice (KDML105). DNA Res 7:93–101.

Lin XH, Zhang DP, Xie YF, Gao HP, Zhang Q (1996) Identifying and mapping a new gene for bacterial blight resistance in rice based on RFLP markers. Phytopathology 86:1156–1159.

Manugistics.1997. Statgraphics Plus for Windows 3.0.Manugistics, Rockville, Maryland, USA.

Marchetti MA, Lai X, Bollich CN (1987) Inheritance of resistance to *Pyriculariaoryzae*in rice cultivar grown in the United States. Phytopathology 77:799–804

Roumen E, Levy M, Nottegham JL (1997) Characterization of the European pathogen population of *Magnaporthe grisea* by DNA fingerprinting and pathotype analysis. European J Pl Path 103:363-371.

Ruengphayak S, Chaichumpoo E, Phromphan S, Kamolsukyunyong W, Sukhaket W, Phuvanartnarubal E, Korinsak S, Korinsak S, Vanavichit A (2015) Pseudo-backcrossing design for rapidly pyramiding multiple traits into a preferential rice variety. Rice 8(7).

Win KM, Korinsak S, Jantaboon J, Siangliw M, Lanceras-Siangliw J, Sirithunya P, Vanavichit A, Toojinda T (2012) Breeding the Thai jasmine rice variety KDML105 for non-age-related broad-spectrum resistance to bacterial blight disease based on combined marker-assisted and phenotypic selection. Field Crops Res 137:186-194.

Yang Z, Sun X, Wang S, Zhang Q (2003) Genetic and physical mapping of a new

gene for bacterial blight resistance in rice. Theor Appl Genet 106:1467–1472.
